# Effects of dietary starch and protein contents on lactation performance, blood metabolites, and methane production of Nili Ravi buffaloes

**DOI:** 10.5713/ab.25.0074

**Published:** 2025-06-24

**Authors:** Hina Tahir, Saima Naveed, Nisar Ahmad, Burhan E Azam, Muhammad Asim Tausif, Sundas Qamar, Saba Anwar, Muhammad Binyameen, Ijaz Hussain, Mubashar Ali, Muhammad Uzair Akhtar, Muhammad Naveed ul Haque

**Affiliations:** 1 Department of Animal Nutrition, University of Veterinary and Animal Sciences, Lahore, Pakistan; 2 Department of Livestock Production, University of Veterinary and Animal Sciences, Lahore, Pakistan; 3 Livestock Experiment Station Bhunikey, Pattoki, Pakistan; 4 Buffalo Research Institute, Pattoki, Pakistan; 5 Department of Livestock and Dairy Development, Lahore, Pakistan; 6 Department of Animal Nutrition, Cholistan University of Veterinary and Animal Sciences, Bahawalpur, Pakistan

**Keywords:** Buffalo, Crude Protein, Interaction, Methane, Milk Yield, Starch

## Abstract

**Objective:**

Imbalanced feeding of protein and energy in ruminants coupled with increasing concerns over low production efficiencies and high livestock emissions intensifies the feeding challenges, especially in buffaloes. This study was planned to evaluate the independent or interactive effects of dietary starch and crude protein (CP) on lactation performance, blood metabolites, and methane production of dairy buffaloes.

**Methods:**

Sixteen lactating multiparous Nili Ravi buffaloes received the following dietary treatments of low (LS) or high starch (HS) content combined with low (LP) or high protein (HP) content in a 4×4 Latin square design: 1) LSLP, 2) LSHP, 3) HSLP, and 4) HSHP. Dietary starch contents were 19.5% and 28.7% in LS and HS diets, whereas CP contents were 8.8% and 10.6% in the LP and the HP diets, respectively.

**Results:**

Although milk yield was not affected by dietary treatments, the HP diet increased milk protein and lactose contents compared with the LP diet in the HS group; however, no such increase was observed in LS group, resulting in a starch×CP interaction. Efficiencies of metabolizable protein and milk nitrogen decreased with the HP diet compared with the LP diet. Metabolic efficiency of metabolizable protein was higher in buffaloes fed the LS diet compared with HS diet. Rumen pH decreased with the HS diet compared with the LS diet in the HP group; however no such change was observed in the LP group. Methane production was increased with the LS diet compared with the HS diet. Contrarily, methane production was decreased in buffaloes fed HP diet compared with those fed the LP diet.

**Conclusion:**

These findings indicated that dietary starch interacted with CP level for milk protein, milk lactose, rumen pH, and methane production of lactating buffaloes. Overall, HS combined with HP content can effectively improve milk protein and lactose contents while reducing the methane emission of lactating buffaloes.

## INTRODUCTION

Balancing of energy and nitrogen in the feeds of production systems have taken great importance due to increasing prices of feed items, animal health concerns and environmental pollution issues [1]. Dietary protein contributes 42% to 50% of the total ration cost [2]. The excessive levels of protein and carbohydrates can result in increased production but decreased conversion efficiency; which can be improved by balancing dairy rations through non-structural carbohydrates to protein ratio [3].

Starch is the main source of energy, which is typically obtained from corn. Dietary starch level has well defined range of 20% to 30% for dairy cows [4]. Increased concentration of starch in the diet of ruminants may lead to lowering rumen pH, which ultimately results into decreased digestion of structural carbohydrate. The quantity of immediate source of energy like non-structural carbohydrates in the rumen has been the major contributor for mitigating nitrogen losses from the dairy animals. Previous studies showed that milk production increased linearly in lactating Nili Ravi buffalo with increased non-structural carbohydrates to crude protein (CP) ratio [5,6].

The improvement in conversion efficiency by balancing non-structural carbohydrates and nitrogen in the diets attributes to the synchronized availability of nutrients leading to the increased microbial yield and overall performance [7]. It was reported that synchronizing carbohydrates and protein may increase nitrogen efficiency [8], ultimately decreasing nitrogen excretion [9]. The milk nitrogen efficiency (MNE) reported for lactating buffalo ranged from 18% to 21% [1,5,10]. However, MNE value for Holstein dairy cows ranged from 26% to 37% [2,11,12]. Previous literature shows that buffalo has lower value of MNE than dairy cow [1,10]. This indicates an opportunity to improve nitrogen efficiency in lactating buffaloes. Volatile fatty acids (mainly propionate and lactate), the carbon skeleton of glucogenic amino acids, and glycerol from the breakdown of triglycerides are the major sources of glucose for ruminants [13,14]. Previous studies have reported that increased dietary starch (15% to 25%) and protein contents (14% to 16%) increased the milk yield and milk protein yield in dairy cows [14]. Similarly, milk protein yield increased when starch content increased from 14% to 22% and protein content from 16% to 18% [15] in dairy cows due to increased blood glucose level, which triggers insulin production and ultimately improves the uptake of amino acids in the mammary gland [16]. Studies related to starch feeding for improving production have been conducted mostly on cows [17,18]. Similarly, a few studies related to protein feeding in buffaloes are available and reported that buffaloes have the potential to perform well on low protein diets compared with dairy cows [1,10]. It was attributed to higher bacterial population, ammonia concentration, better deaminases and salivary recycling of urea, and capacity to maintain positive nitrogen balance with better efficiency of ruminal bacteria to use it during deficiency periods in buffalo compared with cow [19–21]. Additionally, increased dietary starch and starch-protein balance may restrict the hydrogen available for methane production while increasing the microbial protein synthesis, which needs to be explored in lactating buffaloes [22,23]

Differences in digestive physiology, metabolic responses, and milk composition of cow and buffalo, in addition to optimizing the nutrient utilization, improving animal performance, and reducing nutrient wastage are indicative of exploring the effects of dietary starch and protein contents in buffalo diets. However, to the best of the author’s knowledge, limited information is available on the interaction between dietary protein and starch in buffaloes. Moreover, there are growing interests in improving the nutrient efficiencies in buffalo, which is the second largest source of milk and in decreasing the livestock methane emission, which is the second largest greenhouse gas around the world. Thus, it is important to investigate that how dietary starch and protein contents effect the mid-lactating water buffaloes. We hypothesized that dietary starch and protein would have direct and interactive effects on lactation performance, blood metabolites, and methane production of lactating buffaloes. Therefore, the current experiment was aimed to evaluate the interaction and/or independent effects of dietary starch and CP supplies on lactation performance, blood metabolites, and methane production of lactating water buffaloes fed corn silage-based diets.

## MATERIALS AND METHODS

### Animals

The experiment was conducted from February to May 2022 in a tie-stall barn located at the Livestock Experiment Station (LES), Bhunikey, Pattoki, Punjab, Pakistan (73.85°E, 31.02°N, and 186 m altitude). The complete study was conducted in compliance with the ethical guidelines approved by the institutional animal care and use committee, LES Bhunikey, Pattoki, Pakistan (LES-305, 21-02-2022). Sixteen multiparous mid-lactating Nili Ravi buffaloes, with average (mean±SD) milk yield of 9.1±2.1 kg/day, milk fat content of 7.0±0.7%, body weight (BW) of 495±53 kg, parity 2.5±0.5, and 162±15 days in milk were used. Enrolled water buffaloes were individually tied in a ventilated shed with free access to fresh drinking water throughout the day.

### Treatments and experimental design

The buffaloes were divided into four equal groups and each group was randomly assigned four treatments in a 4×4 Latin square design such that within each square buffalo had similar milk production. Treatments were diets, formulated to differ in concentrations of dietary starch and CP: 1) LSLP, low starch and low protein (19.5% starch and 8.8% CP), 2) LSHP, low starch and high protein (19.5% starch and 10.6% CP), 3) HSLP, high starch and low protein (28.7% starch and 8.8% CP), 4) HSHP, high starch and high protein (28.7% starch and 10.6% CP). Total duration of the study was 84 days with each period of 21 days including the first seven days of dietary adaptation in each period. The diets contained 33%, 25%, and 42% corn silage, wheat straw, and concentrate, respectively, on dry matter (DM) basis. Starch content was increased by increasing the ground corn grain and protein content was increased by increasing the soybean meal and protein meal, while primarily replacing the soybean hulls in the diet according to the treatment levels. The dietary forage-to-concentrate ratio was 57:43. The experimental diets were formulated using CNCPS evaluation with CPM-3.0.10 program (Cornell University, Ithaca, NY, USA; University of Pennsylvania, Philadelphia, PA, USA; Miner Institute, Chazy, NY, USA). As the buffaloes were similar in BW and assuming lactation persistency, buffaloes were fed for ad libitum intake for seven days to estimate the dry matter intake (DMI) before the start of experiment, which was subsequently used to formulate and feed for 100% intake. The ingredient and nutrient compositions of the experimental rations are presented in Table 1. Buffaloes were individually fed once in the morning at 09:00.

### Sample collection and analysis

Diets were offered after weighing on a digital weighing balance, while the samples (n = 3) of feedstuff were taken twice during each period for the estimation of DM and the samples were composited for subsequent analysis. Corn silage samples were collected on a weekly basis for DM estimation (#934.01) by drying in a forced-air oven (Memmert 600; Memmert) following the AOAC International [24]. For similar delivery of DM on daily basis, the quantity of corn silage was adjusted every week according to the DM content. These samples were analyzed for nutrient composition following the methods of AOAC International [24], including the CP (#984.13), ash (#942.05), and ether extract (#920.39). Analysis of neutral detergent fiber (NDF; α-amylase+sodium sulfite) and acid detergent fiber (H_2_SO_4_+CTAB) was conducted using a fiber analyzer (ANKOM 2000; ANKOM Technology). Starch concentration in the concentrate samples was estimated from Pakistan Council of Scientific and Industrial Research, Islamabad, according to the methods described by Hodge and Hofreiter [25]. Results of these analysis were used in CNCPS system for experimental total mixed ration formulation. The buffaloes were milked twice daily at 05:00 and 17:00 h. During the first two weeks of each period, milk samples of morning and evening milking were collected on alternate days, while milk samples were collected on daily basis during the last week of each period. The collected samples were analyzed for protein, fat, and lactose contents using Lactoscan Standard milk analyzer (Lactoscan-S; Milkotronic). Samples of blood were taken on the last three days (19–21 d) of each period from the jugular vein [1]. Blood samples were stored and assayed enzymatically by commercial kits (Randox Laboratories) glucose (GL; 2623), triglyceride (TG; TR210), and cholesterol (CH201) concentrations with Rx Monza analyzer (Randox Laboratories). Rumen fluid was sampled from the buffaloes at day 21 of each experimental period using an esophageal polyethylene probe (internal 10 mm and external 14 mm diameters). Samples were collected immediately before the morning feeding to measure ruminal pH. Approximately 0.6 L of rumen fluid was filter through two layers of cheese cloth. After filtration, the pH was measured immediately [26]. The BW of the animals were recorded after milking and before feeding at the start of experiment and at the end of each period. Body condition score (BCS) was recorded at the first and last day of each period by three independent individuals and a median score was noted for each animal following Anitha et al [27].

### Calculations

Non-fibrous carbohydrates (NFC) were estimated according the calculation methods = 100−(CP+NDF+EE+Ash) as described by the NRC [28]. Energy corrected milk (ECM) = (12.95×fat yield)+(7.65×true protein yield)+(0.327×milk yield), 4% fat corrected milk (FCM) = (0.4×milk in kg)+ (15×[fat/100]×milk in kg, 3.4% protein-corrected milk [PCM]) = milk (kg)×0.294% CP, MNE = (N in milk/N intake)×100, milk energy (MkE), and milk nitrogen (MkN) were calculated using the equations presented previously [10]. Gross efficiency of MP = milk protein yield/MP intake and metabolic efficiency of MP = milk protein yield/(MP intake − MP for growth, maintenance, and pregnancy) were calculated as reported in our previous study [29]. Methane production was determined following the equations described by Patra [30].

### Statistical analysis

The PROC MIXED of SAS University Edition was used to analyze the data. Following statistical model was used to analyze the data, with the main effects of starch, CP, starch×CP interaction, and period, while buffalo was considered as a random effect in the model.


(1) $${\text{Y}}_{ijklm}=\mu +{\text{Sq}}_{\text{i}}+{\text{Buff}}_{\text{j}(\text{i})}+{\text{Per}}_{\text{k}}+{\text{S}}_{\text{l}}+{\text{CP}}_{\text{m}}+{\left(\text{S}\times \text{CP}\right)}_{lm}+{ɛ}_{ijklm}$$

Where, Y is the response variable, μ is the overall mean, Buff*_j(i)_* is random effect of buffalo within square, S_l_ is the effect of dietary starch level, CP_m_ is the effect of dietary CP level, Per_k_ is the effect of period, ɛ is the random residual error. The data are presented as least square means with standard error of the means and the differences were considered significant when p≤0.05 and trend towards significance when 0.05<p ≤0.10 using Turkey’s multiple comparison test.

## RESULTS

### Lactation performance

**Figure 1:**
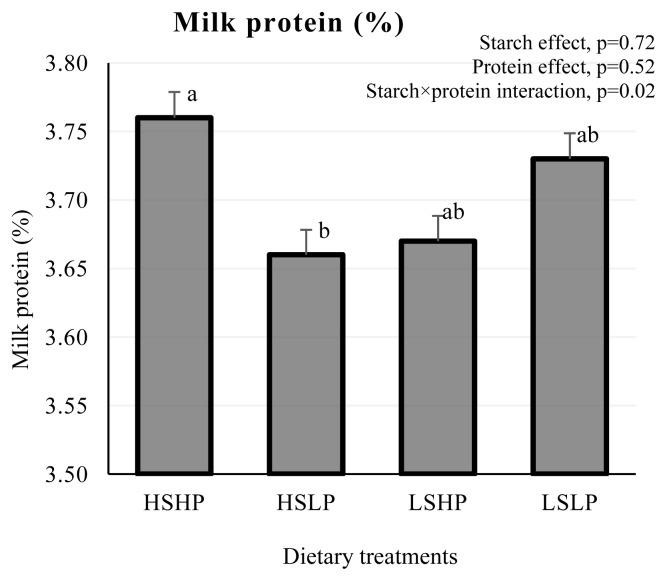
Milk protein content of lactating water buffaloes fed low starch low protein (LSLP; 19.5% starch and 8.8% CP), low starch high protein (LSHP; 19.5% starch and 10.6% CP), high starch low protein (HSLP; 28.7% starch and 8.8% CP), high starch high protein (HSHP; 28.7% starch and 10.6%). ^a,b^ Different lowercase letters above the bars indicate significant difference at p<0.05.

Effects of dietary treatments on lactation performance are presented in Table 2. The DMI did not change across the treatments (p>0.10). Interaction of dietary starch×CP for milk protein content was detected as presented in Figure 1. Interaction of starch×CP indicated that milk protein content increased in HSHP vs HSLP diet from 3.66% to 3.76%, whereas no such increase was observed in LSHP vs LSLP diet (p = 0.02). Milk lactose content increased from 4.96% to 5.10% in HSHP vs HSLP group in contrast to no change detected in LSHP vs LSLP group, resulting in a starch×CP interaction (p = 0.02). Independently, dietary starch (LS; 19.5% vs. HS; 28.7%) and CP levels (LP; 8.8% vs. HP; 10.6%) had no effect on milk yield, milk components, ECM, 4% FCM, 3.4% PCM, milk nitrogen and milk energy (p>0.10). No starch×CP interaction was observed for milk production, ECM, 4% FCM, and 3.4% PCM (p>0.10).

### Production efficiencies

Effects of dietary treatments on nutrient and milk production efficiencies are presented in Table 3. The metabolic efficiency of MP was decreased by 5.81% in water buffaloes of HS group compared with those in LS group (p<0.03). The gross efficiency of MP and MNE decreased in buffaloes fed HP diet by 5.26% (from 0.285 to 0.27) and 14.2% (from 24 to 20.6), respectively, compared with those fed LP diet (p<0.05). No main effects of dietary starch and CP levels were observed on any other production efficiency (p>0.10). The S×CP interaction was not detected for feed and production efficiencies (p>0.10).

### Body weight, body condition score and rumen pH

Effects of dietary starch and CP levels on BW, BCS, and rumen pH are presented in Table 4. An interaction between dietary starch and CP was detected for rumen pH (p<0.05) indicating that rumen pH decreased with HS diet compared with LS diet in HP group, however, no such change was observed in LP group. An increasing trend was observed for BW with increased level of dietary CP (p = 0.07), whereas no effect on BCS and rumen pH was detected in response to dietary starch and CP levels (p>0.10).

### Blood metabolites

Results of blood metabolites are shown in Table 4. No effect of starch and CP levels were observed on the concentrations of plasma glucose, triglycerides and cholesterol levels (p>0.10). The S×CP interaction was not detected for blood metabolites (p>0.10).

### Methane production

Results of methane production in response to dietary starch and CP levels in water buffaloes are presented in Table 5. Increasing the starch level from LS to HS diet, reduced the CH_4_ production (p<0.01). A decrease in CH_4_ production was observed in buffaloes fed HP diet compared with those fed LP diet (p<0.01). The S×CP interaction was not detected for the production of CH_4_ (MJ), CH_4_ (Mcal) and CH_4_ (g/d), however, a decrease of 1.2% and 0.6% in CH_4_ yield (g/kg DMI) was observed when CP level increased in LS and HS diets, respectively, leading to the S×CP interaction (p<0.01). The highest CH_4_ production was noted for LSLP diet as compared to the HSHP diet (p<0.01).

## DISCUSSION

Synchronized supply of protein and energy is the key for optimum rumen functioning and efficient utilization of these nutrients. Exceeding rate of protein degradation than the rate of carbohydrate fermentation results in nitrogen losses. Contrarily, exceeding rate of carbohydrate fermentation than the rate of protein degradation results in reduced microbial protein synthesis with its contribution to increased production of gases [31]. The current study was planned to evaluate the independent and/or interactive effects of dietary starch and CP levels on lactation performance of multiparous mid lactating water buffaloes.

Daily milk yield remained unaffected by dietary starch in agreement with the studies conducted in dairy cows [4,32]. Previously, milk yield was linearly increased by 11% with an increase in dietary starch content from 15% to 25% of DM [17]. Contrary effects were observed on lactation performance with a dietary starch level exceeding 20% in the diet [33]. Average milk yield in our study was 8.63 kg/d and recommended level of starch at this production level in cows is 22% to 26% [28]. No response with increasing starch level in the current study might be attributed to minor differences in the overall energy supply in different treatment groups. However, the inconsistent response of increasing starch levels among various studies could be due to differences in levels of starch in the control and treatment diets, lactation stage of animal, the fermentability of the starch sources, and the effective/forage NDF level in the diet [18]. These variables might partially explain no adequate response of milk yield to varying starch levels (19.5% vs. 28.7%) in the current study.

In the present study, increasing the CP level from 8.8% to 10.6% did not increase milk yield. These findings are in agreement with studies conducted in dairy cows where feeding different levels of CP did not affect the milk yield [34]. On the other hand, previous work in dairy cows reported a quadratic trend in milk yield [11] or an increase when CP level varied from 14.6% to 20.4% [35]. Comparable observations were reported previously by Imran et al [2], where milk yield increased by 5.2% when CP contents increased from 15.2% to 20.9% in the diet of dairy cow. However, milk production decreased in buffalo by increasing dietary CP due to not use of proper CP and energy ratios in the diet [1]. Likewise, the lack of response for milk production in the current study with increasing CP level could be possibly due to the insufficient differences in dietary CP, short duration of treatment, and fixed quantity of feed offered to animals, which might be the limitations of this study. The levels of protein and starch in this study were targeted to match the diets typically offered by the farmers in countries with high buffalo population [36], and to evaluate the distinctive effects of the variations in specific nutrient intake i.e. starch and CP. Additionally, previous literature indicated that protein requirements of buffaloes are less than 12% [37,38] and fall between 9%–11% [39] with no response to diets of CP levels higher than 11% [1]. Possibly, these supplies of starch, CP, and/or their ratios were not close enough in our study to trigger any improvement in milk production in the enrolled buffaloes. Moreover, the buffaloes were in their later stage of mid-lactation, which might also had made it difficult to achieve any dietary effects.

An interaction of S×CP was observed for milk protein and lactose contents in the present experiment. These findings are consistent with the study conducted in dairy cattle [15]. Moreover, starch and protein interaction affects milk composition in dairy cows, primarily by energy-protein balance for the rumen microbes [40]. Additionally, while starch may influence milk protein by altering the metabolism of phenylalanine and tyrosine [41], which might also have supported the increase in milk protein content in the present study. The HSHP diet increased milk protein and lactose contents, which might be attributed to better synchronization of starch and protein for microbial protein synthesis. In the present study, main effect of dietary starch content was not observed for milk composition in agreement with studies conducted in dairy cows [14,15], where feeding 15% to 25% dietary starch levels had no effect on milk fat content and yield. Contrarily, previous studies indicated increased milk protein [4,15] and lactose [4] yields with increasing dietary starch levels. A recent study observed that milk protein and lactose yield increased linearly when starch content increased from 22% to 29% of DM [18]. The amount of lactose produced significantly impacts milk yield, however, in our study milk lactose content was unchanged by main effects of starch and protein. Main effects of dietary protein content on milk composition was not observed in this study. These findings were in agreement with the studies conducted on buffalo [1] or cows [42] reported no effects of varying level of CP on milk composition. In contrast, milk protein increased linearly by increasing dietary CP from 15.2% to 20.9% [2] and 16% to 18%, where milk fat and lactose content remained unaffected [15]. The trivial effects of dietary treatments on milk composition is evident that the supply of starch and protein was sufficient enough to support the milk production of this level, however, at the meanwhile not sufficient to improve the production of water buffaloes at this stage of lactation. It also indicates that the milk production of buffaloes in late stage of mid-lactation can be supported by low dietary CP and starch levels used in this study.

The MNE did not respond to dietary starch level and S×CP interactions. In our study, MNE averaged 22% with feeding of CP level, in agreement with results of previous studies reported in lactating buffaloes, ranging from 18%–21% [1,5,10]. The MNE was decreased with increasing CP supply due to extra nitrogen could not be utilized by microbes of rumen to process for milk nitrogen production [11] or secondly, buffaloes have more efficient urea recycling and have the potential to perform better with low protein diet [43]. Therefore, extra feeding of protein in water buffaloes might be more harmful because their requirements of nitrogen can be satisfied at lower CP in diet as observed in this study and reported previously [44].

An increasing trend was observed in BW by increasing the level of protein in the diet. These results are in agreement with previous reports on buffalo and dairy cattle, where an increase in BW was observed with increase in dietary protein levels [29,45], because of the greater energy movement towards body tissues instead of its utilization to increase the milk production. Contrarily, no change in BW with increasing protein supplies is also reported in dairy buffaloes [1,11]. In the current study, rumen pH was not changed by different dietary starch and protein levels. These findings are in agreement with previous reports where rumen pH of dairy cows was not affected with increasing dietary starch from 22% to 29% [18,26]. Therefore, it can be explained that starch levels in our study (22% to 28%) were in the range, which did not affect pH. Similarly, ruminal pH did not change with different levels of CP, which can be attributed to the low levels of CP used in the diets than required to cause any discernable effects in rumen pH. However, an interaction of S×CP detected for rumen pH might be due to the major treatment difference in starch levels as compared to protein levels. Similar plasma glucose and triglycerides might be attributed to the diets with similar net energy levels, in agreement with previous studies of starch conducted in dairy cows [17,18] and protein feeding in dairy buffaloes [1].

The production of CH_4_ predicted using equation with NDF as a variable supported our initial assumption that a high starch diet would decrease CH_4_ production due to rapid fermentation and enhanced production of volatile fatty acids, especially propionate [46], thereby reducing the CH_4_ production, as reported previously [47]. Reduced hydrogen availability due to 1) shift from acetate to propionate producing microbes with increasing starch content and 2) increased microbial protein synthesis through balanced protein and starch resulting in reduced methane production [22,23]. Interestingly, methane production was reduced with HS diets, whereas the reduction was more pronounced with HSHP diet in this study. Nevertheless, keeping in view the reduced MNE with HP diets and reduced methane with HS diets even with low protein supply is indicative that buffaloes were able to efficiently utilize dietary starch with both low and high protein supplies. Similarly, a trend was observed for lower CH_4_ production (g/d) with the HS diets compared with the LS diets [26]. In addition, starch feeding to ruminants can allow its escape from rumen fermentation, possibly supplying energy to the host animals and avoiding methane synthesis in the rumen. From corn, almost 30% of the starch escape from rumen fermentation and digested in the small intestine. Similarly, an interaction of S×CP showed that feeding low starch diet with high protein level decreased CH_4_ production yield (g/kg DMI) compared to HS diets at high protein level. This could be attributed to efficient rumen physiology of the buffaloes, which includes effective ruminal fermentation [19] and nitrogen utilization [48] due to high ruminal population of cellulolytic, proteolytic, amylolytic, and lipolytic bacteria and fungi [20,49]. This enables buffalo to efficiently use nutrients and produce less methane by digesting cell wall and dietary proteins in a highly efficient way and transform even low nutrient supplies to volatile fatty acids and ammonia [50].

## CONCLUSION

Under the feeding conditions evaluated in the current study, concurrent increases or decreases in dietary starch and protein levels were associated with higher milk protein and lactose concentrations in mid lactating water buffaloes. Diets with low protein content enhanced the gross and metabolic efficiencies of MP utilization and MNE, whereas low starch diets improved the metabolic efficiency of MP. Methane emissions decreased with higher dietary starch and protein levels. No significant interactions were observed between dietary starch and protein supplies on the yields of actual, energy-corrected, 3.4% protein-corrected, 4% fat-corrected milk yields, feed efficiencies, live BW, BCS, and blood metabolites. These findings suggest that high starch and high protein diets can be effectively utilized for mid lactating water buffaloes to improve milk protein and lactose contents, while reducing methane emissions. Future research is warranted to investigate the changes in microbial community, rumen fermentation patterns and kinetics, nutrient digestion, and intestinal flow of microbial protein during different lactation stages in response to varying dietary starch and protein levels, sources, and broader dietary contrasts.

## Figures and Tables

**Table 1:** Ingredients and nutrient composition of the experimental diets

Item		Dietary treatments^1)^		
	
	LSLP	LSHP	HSLP	HSHP
Ingredient (% of DM, unless noted)				
Corn silage	32.7	32.7	32.7	32.7
Wheat straw	25.1	25.0	25.1	25.0
Ground corn grain	8.58	8.58	21.5	21.4
Wheat bran	10.5	8.45	8.46	7.20
Molasses	3.64	3.13	3.47	2.79
Soybean hulls	16.9	14.7	5.42	2.96
Canola meal	0.52	2.88	1.08	3.23
Soybean meal	0.51	2.87	0.86	3.22
Mineral mixture^2)^	0.47	0.47	0.47	0.47
Dicalcium phosphate	0.47	0.47	0.47	0.47
Urea 46%	0.28	0.42	0.33	0.47
Oil^3)^	0.33	0.33	0.14	0.09
Nutrient composition (% of DM)				
DM	54.4	54.5	54.3	54.4
Forage	57.8	57.7	57.8	57.7
Crude protein	8.8	10.6	8.8	10.6
Ash	5.75	5.56	5.23	5.03
Neutral detergent fiber	52.6	51.2	45.5	44.2
Acid detergent fiber	32.2	31.5	27.2	26.4
NFC	31.3	31.2	38.7	38.6
Ether extract	3.13	3.15	3.13	3.12
Predicted nutritive value				
RUP (% CP)	29.8	28.9	30.1	29.0
RDP (% CP)	70.2	71.1	69.9	71.0
Metabolizable protein (g/kg)	76.4	81.1	77.6	83.8
Metabolizable energy (Mcal/kg)	2.20	2.22	2.31	2.34
NE_L_ (Mcal/kg)	1.41	1.43	1.49	1.51
Sugar (% of DM)	4.25	4.34	4.25	4.23
Starch (% of DM)	19.5	19.4	28.7	28.7

1) LSLP = low starch low protein (19.5% starch and 8.8% CP); LSHP = low starch high protein (19.5% starch and 10.6% CP); HSLP = high starch low protein (28.7% starch and 8.8% CP); HSHP = high starch high protein (28.7% starch and 10.6%).

2) Mineral mixture contained 70 kg DCP, 23 kg salt, 5 kg MgSO_4_, 0.7 kg FeSO_4_, 0.5 kg ZeSO_4_, 0.5 kg MnSO_4_, 0.13 kg CuSO_4_, 0.1 kg CoCl, 0.05 kg KI.

3) Oil = mustard oil.

DM, dry matter; NFC, non-fiber carbohydrates; RUP, rumen undegradable protein; RDP, rumen degradable protein; NE_L_, net energy for lactation.

**Table 2:** Milk yield and milk composition of buffaloes fed diets varying in starch and protein levels

	Treatments^1)^					p-value^2)^		
		
Item^3)^	LSLP	LSHP	HSLP	HSHP	SEM	S	CP	S×CP
DMI (kg/d)	14.6	14.6	14.5	14.6	0.02	0.31	0.28	0.74
Yield								
Milk (kg/d)	8.60	8.76	8.54	8.62	0.352	0.49	0.43	0.79
Fat (g/d)	616	635	616	631	27.6	0.82	0.15	0.86
Protein (g/d)	321	321	313	325	13.4	0.73	0.39	0.41
Lactose (g/d)	436	437	425	441	18.3	0.69	0.34	0.42
Milk composition (%)								
Fat	7.19	7.31	7.24	7.33	0.173	0.80	0.42	0.90
Protein	3.73	3.67	3.66	3.76	0.038	0.72	0.52	0.02
Lactose	5.05	4.98	4.96	5.10	0.054	0.81	0.41	0.02
ECM (kg/d)	13.2	13.6	13.2	13.5	0.548	0.67	0.15	0.99
4% FCM (kg/d)	12.7	13.0	12.7	12.9	0.538	0.70	0.16	0.83
3.4% PCM (kg/d)	8.48	8.64	8.30	8.63	0.688	0.79	0.51	0.81
MkN (g/d)	50.3	50.3	49.0	50.8	2.10	0.73	0.39	0.41
MkE (Mcal/d)	9.26	9.16	9.12	9.42	0.381	0.73	0.13	0.78

1) LSLP = low starch low protein (19.5% starch and 8.8% CP); LSHP = low starch high protein (19.5 % starch and 10.6 % CP); HSLP = high starch low protein (28.7% starch and 8.8% CP); HSHP = high starch high protein (28.7% starch and 10.6%).

2) Main effects of dietary treatments: S = starch; CP = protein; S×CP = starch by protein interaction.

3) ECM = energy corrected milk, 4% FCM = fat corrected milk, 3.4% PCM = protein corrected milk, MkN = milk nitrogen, MkE = milk energy were calculated according to the equations presented previously [10].

SEM, standard error of the mean; DMI, dry matter intake.

**Table 3:** Feed and milk production efficiencies of buffaloes fed diets varying in starch and protein levels

	Treatments^1)^					p-value^2)^		
		
Item	LSLP	LSHP	HSLP	HSHP	SEM	S	CP	S×CP
Feed efficiency^3)^	0.59	0.60	0.59	0.59	0.021	0.45	0.40	0.74
ECM: DMI^4)^	1.60	1.62	1.38	1.59	0.239	0.58	0.60	0.69
4% FCM: DMI^4)^	0.87	0.90	0.87	0.88	0.036	0.65	0.13	0.79
3.4% PCM: DMI^4)^	0.58	0.59	0.57	0.59	0.061	0.90	0.75	0.92
Gross efficiency of MP^5)^	0.29^a^	0.27^ab^	0.28^ab^	0.27^b^	0.012	0.14	0.02	0.63
Metabolic efficiency of MP^6)^	0.45^a^	0.41^b^	0.42^ab^	0.39^b^	0.019	0.03	0.01	0.66
MNE (%)^7)^	24.3^a^	20.5^b^	23.7^a^	20.7^b^	0.95	0.62	0.01	0.44
MkN: MkE (g/Mcal)^8)^	5.43	5.32	5.35	5.39	0.100	0.99	0.64	0.33

1) LSLP = low starch low protein (19.5% starch and 8.8% CP); LSHP = low starch high protein (19.5% starch and 10.6% CP); HSLP = high starch low protein (28.7% starch and 8.8% CP); HSHP = high starch high protein (28.7% starch and 10.6%).

2) Main effects of dietary treatments: S = starch; CP = protein; S×CP = starch by protein interaction.

3) Feed efficiency = milk production/dry matter intake.

4) Ratio of energy corrected milk (ECM), 4% fat corrected milk (FCM), and 3.4% protein corrected milk (PCM) with dry matter intake (DMI).

5) Gross efficiency of metabolizable protein (MP) = milk protein yield/MP intake [29].

6) Metabolic efficiency of MP = milk protein yield/(MP intake − MP for growth, maintenance, and pregnancy) [29].

7) Calculated from MNE = milk nitrogen efficiency = N in milk/N intake [10].

8) MkN: MkE = milk nitrogen: milk energy [10].

a,b Means within a row with different superscripts differ significantly (p<0.05).

SEM, standard error of the mean.

**Table 4:** Body weight (BW), body condition score (BCS), rumen pH and blood metabolites with different starch and protein levels

	Treatments^1)^					p-value^2)^		
		
Item	LSLP	LSHP	HSLP	HSHP	SEM	S	CP	S×CP
BW (Kg)	549	556	544	551	14.5	0.18	0.07	0.92
BCS	3.30	3.28	3.33	3.28	0.090	0.76	0.55	0.76
Rumen pH	6.78	6.80	6.79	6.74	0.018	0.24	0.39	0.05
Glucose (mg/dL)	78.2	80.1	79.5	78.5	1.618	0.92	0.77	0.36
Cholesterol (mg/dL)	109	114	114	115	3.958	0.44	0.38	0.65
Triglycerides (mg/dL)	114	111	116	119	3.914	0.19	0.92	0.54

1) LSLP = low starch low protein (19.5% starch and 8.8% CP); LSHP = low starch high protein (19.5% starch and 10.6% CP); HSLP = high starch low protein (28.7% starch and 8.8% CP); HSHP = high starch high protein (28.7% starch and 10.6%).

2) Main effects of dietary treatments: S = starch; CP = protein; S×CP = starch by protein interaction.

SEM, standard error of the mean; BW, body weight; BCS, body condition score.

**Table 5:** Methane (CH_4_) production with different starch and protein levels

	Treatments^1)^					p-value^2)^		
		
Item	LSLP	LSHP	HSLP	HSHP	SEM	S	CP	S×CP
CH_4_ (MJ)^3)^	14.8^a^	14.7^b^	14.1^c^	14.0^d^	0.02	<0.01	<0.01	0.55
CH_4_ (Mcal)^4)^	3.55^a^	3.51^b^	3.38^c^	3.34^d^	0.003	<0.01	<0.01	0.55
CH_4_ (g/d)^5)^	248^a^	245^b^	236^c^	234^d^	0.4	<0.01	<0.01	0.55
CH_4_ (g/kg DMI)^6)^	17.0^a^	16.8^b^	16.1^c^	16.0^d^	0.01	<0.01	<0.01	<0.01
CH_4_ (g/kg milk)^7)^	30.0^a^	29.1^ab^	29.0^ab^	28.3^b^	1.57	0.03	0.05	0.75

1) LSLP = low starch low protein (19.5% starch and 8.8% CP); LSHP = low starch high protein (19.5% starch and 10.6% CP); HSLP = high starch low protein (28.7% starch and 8.8% CP); HSHP = high starch high protein (28.7% starch and 10.6%).

2) Main effects of dietary treatments: S = starch; CP = protein; S×CP = starch by protein interaction.

3) CH_4_ (MJ) = methane production in mega joule = (−0.436+0.678×DMI+0.697×NDF intake).

4) CH_4_ (Mcal) = methane production in mega calorie = CH4, MJ/4.18.

5) CH_4_ (g/d) = methane production in gram per day = (0.671/40)×methane production in mega joule×1,000.

6) CH_4_ (g/kg DMI) = methane yield in gram per kg of DMI = CH_4_, g/d/DM.

7) CH_4_ (g/kg milk) = methane intensity in gram per kg of milk.

a–d Means within a row with different superscripts differ significantly (p<0.05).

SEM, standard error of the mean; DMI, dry matter intake; NDF, neutral detergent fiber.
